# Selection of the Most Scenic Viewpoints on an Island Based on Space–Time Perception: The Case of Nan’ao Island, China

**DOI:** 10.3390/ijerph19031309

**Published:** 2022-01-24

**Authors:** Tongyan Zhang, Shengrui Zhang, Yingjie Wang, Hu Yu, Hongrun Ju, Hanyun Xue

**Affiliations:** 1 Management College, Ocean University of China, Qingdao 266100, China; Zhangtongyan@ouc.edu.cn (T.Z.); xuehanyun2021@163.com (H.X.); 2 Institute of Geographic Sciences and Natural Resources Research, Chinese Academy of Sciences, Beijing 100101, China; wangyj@lreis.ac.cn (Y.W.); Yuhu@igsnrr.ac.cn (H.Y.); 3 School of Tourism and Geography Science, Qingdao University, Qingdao 266071, China; juhr@radi.ac.cn

**Keywords:** space–time perception, scenic viewpoint evaluation, island tourism, GIS method, China

## Abstract

Selecting the most scenic viewpoints in an island forest park can provide a scientific basis for island ecotourism planning. In this paper, considering the influence of climatic conditions on sightseeing, landscape factors, accessibility factors, and seasonal change factors are selected from the perspective of time and space to analyze the landscape spatiotemporal characteristics, and the construction of a landscape spatiotemporal perception evaluation model and the analysis of influencing factors are carried out. The results show that the evaluation model for landscape spatiotemporal perception factors can quantitatively describe tourists’ comprehensive perception of a landscape in different regions and time periods in ecotourism areas, and can identify the spatiotemporal characteristics of landscape perception. Case studies preliminarily prove the feasibility of the model and calculation process. This method provides a strong reference for the development and planning of island tourism, especially providing new ideas and methods for the design of sightseeing routes in the development and planning of small-scale scenic viewpoints, which can enrich island tourism planning.

## 1. Introduction

The selection of an island’s most scenic viewpoints is an important way to design tourism routes and provide tourists with a high-quality tourism experience, especially for the development of island tourism resources. The scientific choice of scenic island viewpoints directly affects the overall spatial organization and optimization of a tourism area. Scientific selection of the most scenic viewpoints can meet the practical requirements of tourists, thereby providing a better tourism experience. Lookout point selection involves a lot of complexity, however, involving not only the viewing subject, but also the choice of location, landscape type, terrain conditions, and factors such as comprehensive influence [1]. Islands’ uniqueness complicates the gradation and diversity of landscape layout, so the study of scenic island viewpoints and their selection has important practical significance.

Tourism destination planning is an essential element of regional tourism systems; in the island tourism destination category [2], the main research is still in the basic descriptive study phase, focusing on the tourism development environment [3], strategy [4], and mode of empirical summary [5,6]. There is not yet any quantitative research on choosing scenic viewpoints. In the early stages, the selection of scenic viewpoints mostly depends on the intuitive judgment of planners or architects, and lacks a reasonable optimization selection process framework. The 3S (RS, GIS, GPS) technology provides more scientific and accurate destination planning optimization. Related research has been conducted on landscape quality measures [7], the visual landscape structure [8], the layout structure of tourism destinations, tourism line organization, the distribution of facilities, and construction sites. In terms of natural tourism sites, Nijhuis (2015) [9] selected scenic mountain viewpoints based on the view range analysis of mountain landscapes and the scores of different scenic source points given by experts. Ma (2020) [10] explored a new method of tour route planning for small-scale hilly and mountainous scenic areas; introduced multiple big data sources such as natural geographic factors, Google Earth photo sharing data, and mobile phone signaling data to select natural and cultural scenic viewpoints; and identified tourist routes based on multidimensional visual landscape evaluation. On the pretext of selecting ecological safety zones and tourism corridors, Ginzarly (2019) [11] further screened out the most scenic viewpoints through a landscape view analysis. There are few studies on the selection of scenic viewpoints in cultural tourism destinations, and only the most scenic viewpoints are extracted from traditional villages, with Yin (2015) [12] taking Kaiping Diaolou as an example. During the selection process, it is important to build a landscape evaluation model through a fuzzy analytic hierarchy process (AHP), based on the visual landscape sensitivity perception model range, the best viewing distance, the best viewing azimuth horizon perception influence factor, the integration of ecological awareness, traffic accessibility, and the suitability of the terrain for habitation [13,14,15]. At present, there is a lack of systematic selection methods for scenic island viewpoints, and the dimension is relatively singular, ignoring the dynamic changes in the island landscape over time. For offshore islands, the sunrise and sunset viewing value is an important factor in tourism development evaluation [16]. The existing research fails to include the time dimension to maximize the exploitation of island tourism resources, so it is urgent to strengthen the research on methods for selecting the most scenic island viewpoints.

Taking Nan’ao Island as an example, this paper combines landscape topographic characteristics, accessibility, and environmental changes with landscape perception, and introduces factors such as slope, relief, landscape view, accessibility, and seasonal change to quantitatively calculate tourists’ landscape perception from the perspectives of time and space. Then, the scientific method of optimal scenic viewpoint location and route optimization is discussed from the perspective of tourists’ perception of time and space differentiation.

## 2. Research Methods and Data Sources

### 2.1. Overview of the Study Area

Nan’ao Island is the most beautiful island and coastline in Guangdong Province, integrating islands, mountains, coastlines, and offshore waters, with rich natural and cultural tourism resources. It is an ideal place to carry out coastal leisure tourism, marine island ecological science popularization, natural education, and historical and cultural experiences, and has the potential to create a national AAAAA tourist attraction. Nan’ao Island has a high degree of ecological and ecosystem diversity, including terrestrial (natural and artificial), coastal, and marine ecosystems, and forest parks. The existing scenic area includes the Guicheng scenic area and the Changshanwei Garden; a new scenic area is in Shantou City, where Gaozhang Mountain is the highest peak at 587.1 m. The research area is the only island crossing the Chinese mainland along the Tropic of Cancer, so it is a crossroads for international migratory birds, a breeding ground and habitat for endemic birds in the northernmost part of the world, the earliest maritime exchange and barter node in East Asia to participate in the process of global trade, and a major coastal defense center that has controlled the coastal defense affairs of eastern Guangdong, southern Fujian, Taiwan, and the Penghu Islands in Chinese history.

### 2.2. Research Design

#### 2.2.1. Preliminary Selection of Scenic Viewpoints

**Figure 1:**
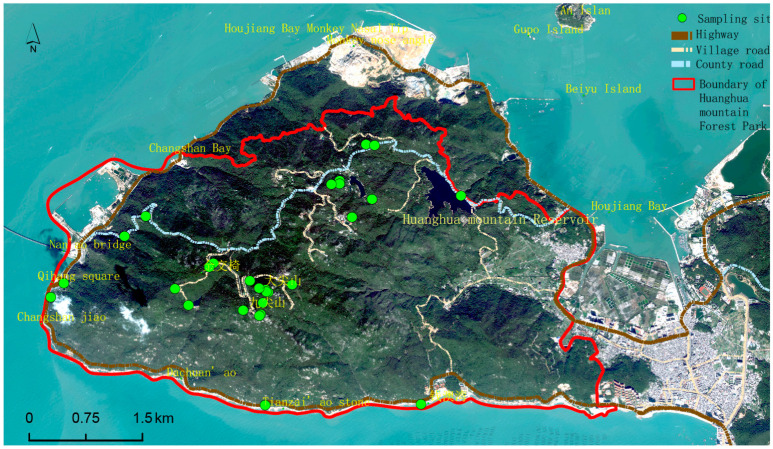
Primary location of sample plots based on terrain characteristics.

Based on an analysis of topographic characteristics of the study area, this paper establishes preliminary selection criteria for scenic viewpoints from four angles: elevation, relief, slope, and aspect (Table 1). According to the mountain elevation, the scenic viewpoints are divided into three levels: top scenic viewpoints, hillside scenic viewpoints, and foothill scenic viewpoints. The characteristics of a scenic viewpoint on the top of a mountain are a wide field of vision, noteworthy scenery, and a wide field of vision. Most of the scenic viewpoints on the mountainside are details of the mountain landscape and medium-sized landscape, which can be viewed from a variety of perspectives. The scenic viewpoints in the foothills are mainly viewed from above, so visitors have a general understanding of the mountains they see and have an impulse to explore them [17]. Based on tourism psychology, human visual perception habits and scenic area planning specifications [18] are assessed using ArcGIS 10.2 software to map the elevation of 28 sample areas in the study area, mainly at Dajianshan Mountain, Jiudajian Mountain, Huanghua Mountain, Changshanwei Wharf, the Houyandun site, and the Dachuan’ao area (Figure 1).

#### 2.2.2. Construction of a Landscape Temporal and Spatial Perception Model

##### **Measurement of Visual Landscape Quality Indicators** 

(1) Open field of vision

Open field of vision refers to the total visual area of all areas seen by the observer from one or more scenic viewpoints. The larger the visual area, the better the visual landscape quality and the wider the coverage. The visual field openness of the island is mainly affected by the coastline curve; the visual field of the convex part of the coastline is more open, and the visual field of the concave part of the coastline is more closed. The height of scenic viewpoints is also an important factor influencing the degree of visual field opening. The higher the scenic viewpoint, the larger the visual field, the fewer occluded objects in the visual field, and the higher the spatial visual pleasure.

The open area in this paper can be divided into mountains and seas, which is the area of all mountains and waters seen from one or more observation points. The open area of the mountain refers to the total area of the mountain that the observer can see in the scenic viewpoint, and the open area of the water refers to the area of the sea that the observer can see from the scenic viewpoint.

(2) Visual richness

The aesthetic depth of a landscape that an observer can see in a certain direction may refer to different upper body levels or other landscape depth. When viewing the scenery from a relatively fixed viewpoint, the scenery itself forms a visual hierarchy due to the difference in distance [23]. The research area is located on an island, surrounded by the mountains as well as the sea, with a long view of sea-level marine buildings and other islands. The middle view is the bedrock and sedimentary geomorphic landscape distributed along the coastline, and the close view is the mountain forest vegetation landscape, rock landscape, and water landscape.

(3) Number of landmark landscapes

The landmark landscape refers to the representative and unique landscape of a region, often referring to the landmark scenic viewpoints of a city. The type of landmark landscape can be natural or cultural. It can be point-like, plane-like, or line-like in space. The number of landmark landscapes refers to the number of landmark scenic viewpoints seen from candidate sites.

According to the characteristics of the natural environment in Nan’ao County, 133 landmarks of the island were selected, including the sea surface, tidal surge and wave breaking phenomena, strange and pictographic stones, a sea bridge, the Tropic of Cancer marker, farmland, a reservoir, a lake, temples, etc.

##### **Observation Site Accessibility Measurement** 

The accessibility of scenic viewpoints requires a quantitative evaluation of the degree of convenience or difficulty in reaching scenic viewpoints, which is used as the basis to judge the feasibility. This article uses the weighted distance cost method to measure accessibility to forest park entrances as a starting point, considering terrain (slope), objects (such as roads and rivers), the motivation of visitors to overcome resistance, time, and distance. The measure of the time cost distance is the traffic access time; its value should be the reciprocal of the traffic speed, and the formula is as follows:

(1) $$\cos t=\frac{1}{v}\times 60,$$

where v is the set speed of various space objects, in km/h; cost is the time cost, in min/km. The specific value of v is set according to the industrial standard of the People’s Republic of China (Highway Engineering Technical Standard (JTG801-2003)) and the actual traffic situation of different grades of highway in Nan’ao County. The average driving speed of all grades of highway traffic in the study area is set, and the time cost value is calculated in minutes (Table 2). In addition, the study area in this paper is mountainous and hilly, and accessibility is limited by the terrain, so the slope and relief of the terrain are included in the time cost value (Table 3).

##### **Best Viewing of Sunrise and Sunset in Different Seasons** 

(1) Sunrise and sunset azimuth calculation

The research area in this paper is Nan’ao Island, located between 116°53′ and 117°19′ east longitude and 23°11′ and 23°32′ north latitude. The Tropic of Cancer (23°26′ N) crosses the island. The summer solstice occurs on 21 June or 22 June every year, when the sun directly hits the Tropic of Cancer, and is the longest day in the northern hemisphere. At 12:13 p.m., there will be a “pole without a shadow” on the Tropic of Cancer. In the northern hemisphere, the position of sunrise is observed in the northeast in the summer and the sunset in the northwest. In winter, the sunrise is observed in the southeast and the sunset in the southwest. When the direct point of the sun is located at the Tropic of Cancer (i.e., the summer solstice), the sun rises at 25°42′ east by north and sets at 25°42′ west by north, which is the viewing area. When the direct point of the sun is located at the Tropic of Capricorn (i.e., the winter solstice), the sun rises at 25°42′ east by south and sets at 25°42′ west by south, which is the viewing area. The best viewing area is one where one can see both the sunrise and sunset, while a general viewing area is one where one can only see the sunrise or the sunset.

Solar altitude angle:

(2) sinHs=sinφ·sinδ+cosφ·cosδ·cost.

In Equation (2), Hs represents the solar altitude angle, φ represents the geographical latitude, δ represents the solar declination, and t represents the time.

Solar azimuth:

(3) cosAs=(sinHs·sinφ−sinδ)/(cosHs·cost).

In Equation (3), Hs represents the solar altitude angle, φ represents the geographical latitude, and δ represents the solar declination.

(2) Best viewing point metric

In this study, 23°26′ N and 117° E were used as location standards to calculate the solar altitude angle and azimuth angle during sunrise and sunset. Terrain shadow analysis was used to obtain the viewing area, and the sample plots that did not fall into the viewing area were set to 0.001 for the optimal viewing azimuth score. To evaluate and measure the sample land falling into the ornamental area, the calculation formulas are as follows:

(4) $$F(x_{i})=\sum_{i=1}^{4}P(x_{i})$$

(5) $$P(x_{i})=\frac{1}{N_{i}}.$$

In Equations (4) and (5), *F*(*x_i_*) is the score value of the best viewing azimuth at the drop sample site *x*, and *x_i_* is the *i* season sample site. *P*(*x_i_*) is the probability of the plots falling into the ornamental area in season *i*, and *N_i_* is the total number of plots falling into the ornamental area in season *i*.

##### **Landscape Temporal and Spatial Perception Evaluation Model** 

The spatiotemporal impact factors of the above scenic viewpoints are standardized, and the calculation model of site selection suitability of scenic viewpoints in ecotourism is established. The calculation formula is as follows:

(6) $$S=w_{1}\times Q+w_{2}\times B+w_{3}\times T.$$

In Equation (6), S is the site selection suitability of the sample site, Q is the visual landscape quality of the sample site, B is the accessibility of the sample site, and T is the score value of the best viewing orientation of the sample site. $w_{i}$ is the weight value of the spatiotemporal factor, which is calculated by using Yaahp software (V9.1) combined with the analytic hierarchy process and expert scoring method (Table 4).

#### 2.2.3. Network Analysis Model Construction

A network analysis model is an abstract representation of various network systems in the real world, mainly composed of a set of basic elements such as chains, nodes, central source points, and resistances connected to each other according to a certain topological relationship [24]. In this study, the park entrance and scenic viewpoints in the study area were taken as the basic elements of the network analysis model, and ArcGIS10.2 software was used to build the network model. Through the analysis of the lowest-cost path, the spatial path with the lowest obstacle cost between nodes in the network was extracted to generate the optimal viewing route.

### 2.3. Data source and Processing

The remote sensing images (GF-2 in January 2019, spatial resolution 0.8 m) used in this study were provided by the China Resources Satellite Application Center, and have been orthocorrected through image fusion and atmospheric correction. Topographic data included a 1:5000 topographic map of the study area (provided by the Natural Resources Bureau of Nan’ao County). ArcGIS10.2 software was used to transform the contour vector map into a digital elevation model (DEM) with a spatial resolution of 2.5 m, and we vectorized the boundary of main roads and scenic viewpoints. The observation data and landscape analysis data used in this paper were all from the tourism resource survey data, and a total of 1378 tourism resource points were collected. The survey was conducted in accordance with the Chinese National Standard Tourism Resources Classification Rules (GBT 18972-2017), and the data came from the Master Plan of Nan’ao Island National Forest Park in Guangdong Province (2018–2027), the second geographical name census database, high-resolution remote sensing images, and a field survey from 2019. The collected attribute information of tourism resources included location, type, nature and characteristics, surrounding environment, and protection and development conditions. In addition, the expert scoring method was used to score the landscape value of tourism resources. In this study, the point data of tourism resources were used as the sample set of the most scenic viewpoints, and the location, type, nature, and characteristics of attribute information, surrounding environment, protection and development conditions, and quality grade were considered.

## 3. Results and Analysis

### 3.1. View Range Analysis of Scenic Viewpoints

#### 3.1.1. Analysis of Visual Area

**Figure 2:**
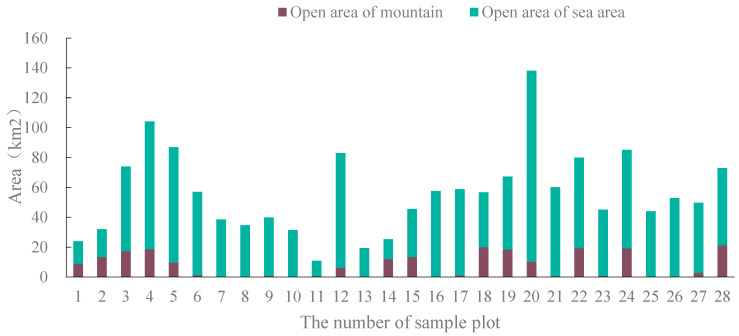
Distribution of open area in the sight area of the sample plot.

According to the field view analysis of the study area, the visible landscape area, mountain area, and sea area of the sample area were obtained statistically, as shown in Figure 2. Sample plots 4 and 20, with a visual area greater than 100 km^2^, are on Dajian Mountain. Sample plots 3, 5, 12, 22, 24, and 28 (70–100 km^2^ plots) are mainly located in Houyandun and on Huanghua Mountain to the north and Dajian Mountain and Jiujian Mountain to the south. Sample plots 11 and 13, with a visual area of less than 20 km^2^, are in Gouguguizai, a southern pit. The larger the visible mountain area and sea area, the better the view of the sea and mountains from the sampling position. The study area is surrounded by a vast sea area, and the observed sea area at the sample sites accounts for a high proportion. Sites 6–11 of the sample sites are only suitable for sea observation, mainly at Changshanwei Wharf, Tianzai Geopark, and Guzai Pit in the southwest coast area.

#### 3.1.2. Analysis of Visual Richness

**Figure 3:**
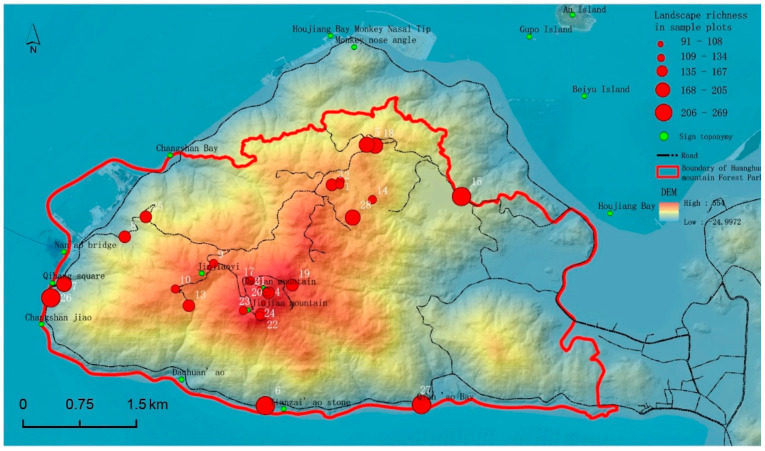
Hierarchical distribution of landscape richness in sample plots.

Aiming at the analysis of visual richness, this study took the distance (10 km) of the contour clearly discernable by human eyes as a buffer to calculate the depth of the scenery that could be seen in a certain direction and graded the visual richness of 28 sample sites (Figure 3). As can be seen from the figure, the observation points with rich landscape levels are mainly distributed on the coast and lakeshore, located at the middle and low altitudes, i.e., upward viewing, so it is easy to emphasize the height and unusual shapes of the scenery. One can see a close view of the sea, a middle view of the mountain and forest, and a distant view of the exposed rock on the mountain top, with rich visual landscape levels. The top of the mountain is at a lower level and higher altitude, which makes it an overlook that gives people a feeling of openness. As the study area is shaped like a triangle, the distance from the peak to the coast is only 1–3 km, with a large height difference. Standing on top of the mountain, people can only see the close mountain and the distant sea, so the visual landscape hierarchy at the top of the mountain is poor.

#### 3.1.3. Analysis of Marked Landscape

**Figure 4:**
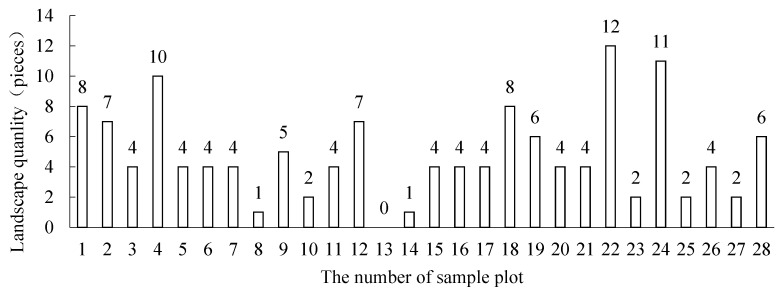
Number of landmark landscapes observed in sample plots.

As for the number of marked landscapes observed in the sample plot, the more marked landscapes there are, the higher the ornamental value of the sampling location is, as shown in Figure 4. The plots with numbers more than 10 include 4, 22, 24; plots with numbers between 5 and 10 include 1, 2, 5, 12, 18, 19, 28; plot 13 has no marked landscape within the visual range.

Through the type analysis of the marked landscape, there are 12 observed landscape types, as shown in Table 5. Forest farm, farmland, peculiar and pictographic stone, and reef landscape can be observed in 40–50% of the sample plots in the study area. The phenomenon of sea surface breaking waves and lake landscape are difficult to observe due to the influence of climate and topography.

### 3.2. Accessibility Analysis of Scenic Viewpoints

**Figure 5:**
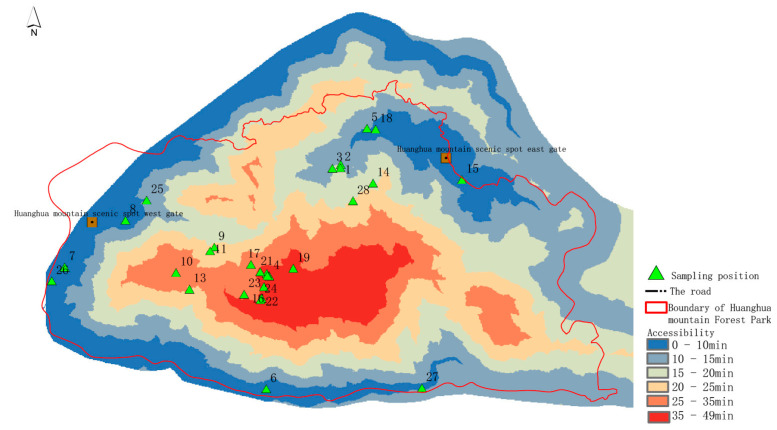
Accessibility analysis of sample plots.

Scenic viewpoints should be considered in landscape architecture, especially designing tourist facilities. Scenic viewpoints should be easy for tourists to reach. Therefore, accessibility is a factor that needs to be considered in site selection. The better the accessibility, the easier it will be for tourists to reach these sites, and it is also conducive to the centralized management of scenic viewpoints. In this study, two entrances and exits were selected to analyze the accessibility of the research area. Using 10 min, 15 min, 20 min, 25 min, 35 min, and 49 min as the standard, the accessibility of the sample plot was divided into six time periods, and the results are as shown in Figure 5. The distribution difference of accessibility is obvious in scenic viewpoints. Relatively poor accessibility is seen in the Dajian Mountain area, where the altitude is higher and the slope is steeper, so reaching it takes up to 49 min. The areas with better accessibility are distributed along the coast, with good roads and better traffic conditions.

### 3.3. Diurnal Analysis in Different Seasons

**Figure 6:**
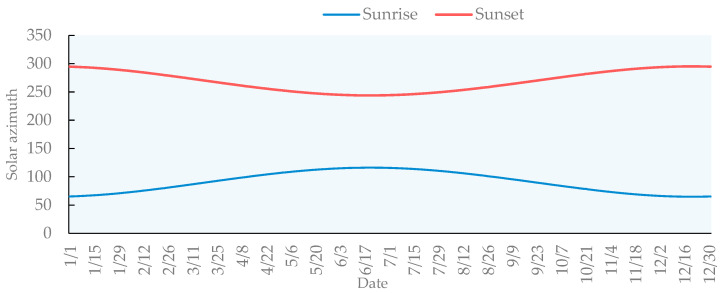
Azimuth angle of sunrise and sunset throughout the year in the study area.

This paper analyzes the time and location of sunrise and sunset observations for the whole of 2020. The azimuth of sunrise is 71.7592–106.866° in spring, 107.4–116.175° in summer, 74.586–109.772° in autumn, and 64.7317–74.236° in winter. The sunset azimuth is 253.104–288.3146° in spring, 243.916–252.978° in summer, 250.195–285.4419° in autumn, and 285.7903–295.2699° in winter. The earliest sunrise is on 8 June at 5:24:47 with a solar azimuth of 115.591°, and the latest sunrise is on 14 January at 6:55:42 with a solar azimuth of 67.192°. The earliest sunset will be at 17:24:54 on 28 November with a solar azimuth of 293.152°, while the latest sunset will be at 19:02:29 on 2 July with a solar azimuth of 244.475°. The annual trend of sunrise azimuth first increased and then decreased, and the minimum value appeared on 14 June. The trend of the sunset azimuth first decreased and then increased, and the maximum value appeared on 20 June (Figure 6).

As can be seen from Table 6, the best sunrise viewing period is from 5 a.m. to 7 a.m., and the sunset viewing period is from 5 p.m. to 7 p.m. The number of sample plots falling into the viewing area is 21, among which 12 are for sunrise viewing in spring and 10 are for sunset viewing. In summer, eight sites were used to watch the sunrise and six for the sunset. In autumn, six sites were used to watch the sunrise and five for the sunset. In winter, there were five sites used for sunrise viewing and six for sunset viewing. Sunset observation points 18, 1, and 2 appeared with high frequency, while sunset observation points 14, 21, 25, and 26 appeared with high frequency.

**Figure 7:**
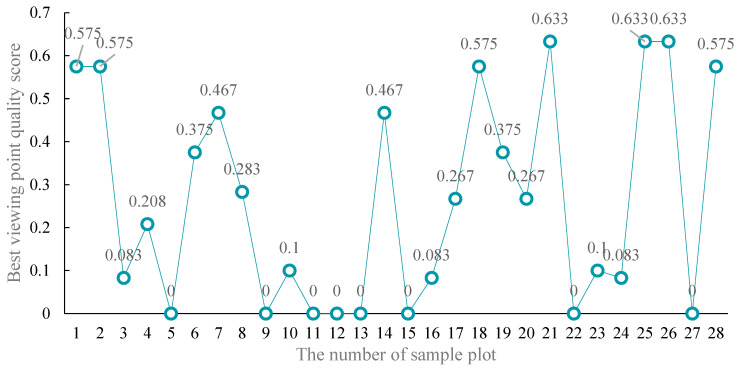
The score value of the best ornamental point quality in the sample sites.

The ornamental quality score F of each sample site was calculated by the measurement model for the most scenic viewpoint (Equation (4)) (Figure 7). Sample sites 21, 25, 26, 1, 2, 18, and 28, with an F score greater than 0.5, were the most scenic viewpoints, mainly located in the area of Dajian Mountain and Bengkan Mountain. The sample sites with an F score between 0.1 and 0.5 included 7, 14, 6, 19, 8, 17, 20, 4, 10, and 23, which are the secondary ornamental sites, mainly located on Jiujian Mountain and the Changshanwei area. The sample sites with an F score between 0 and 0.1 included 3, 16, and 24, which are mainly located on Jiujian Mountain. Plots 5, 9, 11, 12, 13, 15, 22, and 27 are not the best sites for viewing the sun. They are mainly located in Yandun Beacon Tower Ruins, Guizaikeng Trail, and Qian’ao Bay.

### 3.4. The Best Site Selection for Scenic Viewpoints

Through field investigation, sample pictures, and spatial analysis, we selected 28 sample observation points and compared them through a horizon, accessibility, and best viewing azimuth and time comparison analysis and the removal of adjacent sampling locations. The top 13 plots were calculated as the most scenic viewpoints of Huanghua Mountain Forest Park by using the spatiotemporal perception factor evaluation model. The plots are 2, 3, 5, 6, 7, 15, 18, 19, 20, 21, 25, 26, and 28. The 13 most scenic viewpoints were analyzed by hierarchical clustering according to the open area of visual field, visual field richness, the number of marked landscapes, accessibility, and the best viewpoints. The different visual landscapes were divided into four types: those best suited for enjoying the sea view and the sunrise and sunset, those best suited for watching the mountains and the coast, those best suited for watching mountains and lakes, and those best suited for sighting unusual or pictographic stones and vegetation (Table 7).

### 3.5. Island Scenic Route Layout

**Figure 8:**
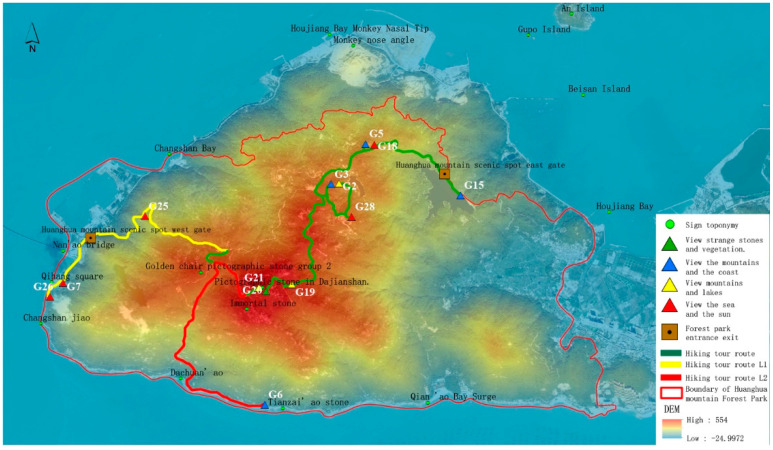
Walking tour route in Huanghua Mountain scenic area.

**Figure 9:**
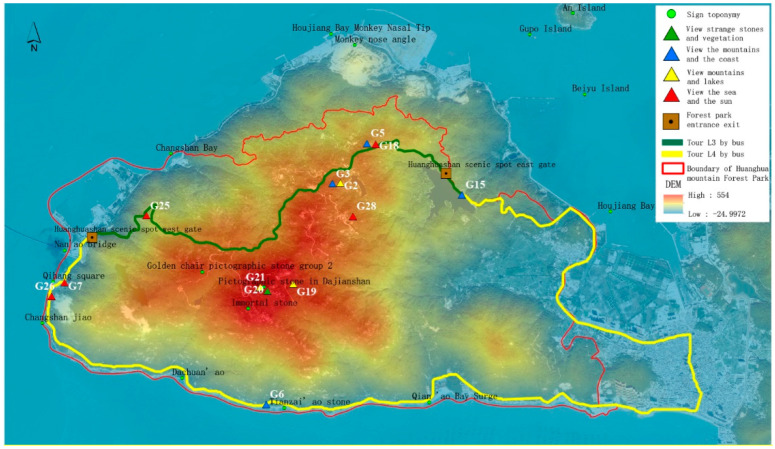
Bus tour routes in Huanghua Mountain scenic area.

According to the spatial distribution of the 13 most scenic viewpoints in Huangshan Forest Park, the lowest spatial cost data of the study area were created based on existing roads, landforms, and different land types. Two entrances and exits of the scenic viewpoints were selected and the viewing routes of Huanghua Mountain scenic viewpoints were generated using the ArcGIS least cost path analysis method. Combined with the existing traffic system and development status of the scenic viewpoint, a scenic route is generated and divided into a walking tour route and a bus tour route, as shown in Figure 8 and Figure 9. As can be seen from Figure 8, walking tours can be divided into two routes, L1 and L2. L1 is from the east gate of Huanghua Mountain to the west gate of Huanghua Mountain, and L2 is from Tianzai Geopark and Guizai Keng Valley to the east gate of Huanghua Mountain. L1 line: east gate of Huanghua mountain—G18 (sunrise)—G3 (forest)—G2 (lake)—G19 (farmland)—G20 (strange rocks)—G25 (sunset)—west gate of Huanghua mountain; line L2: start from Tianzi Geopark G6 (sunrise)—G20 (rocks)—G19 (farmland)—G28 (islands)—G2 (lake)—G3 (forest)—G18 (sea)—east gate of Huanghua Mountain.

As can be seen from Figure 9, the bus tour can be divided into L3 and L4, which are circular island routes that take in different types of coastal landscapes. The L3 line starts from the west gate of Huanghua Mountain and goes along provincial road 336 to the north road to the east gate; along the way, one can enjoy the northern headland and coastal landscape. Then, from the east gate, the route goes along county road 057 through the G5 and G25 scenic viewpoints, and finally back to the west gate. The L4 line starts from the west gate of Huanghua Mountain and goes along provincial road 336 to the south road to the east gate; along the way, one can enjoy the bedrock and sandy beach along the south coast landscape. Then, from the east gate, the route goes along county road 057 through the G5 and G25 scenic viewpoints, and finally back to the west gate.

## 4. Discussion

Due to its superior geographical location, the island has natural landscapes such as coast, mountains, and forests, and animals, meteorology, etc. These natural landscapes themselves have the characteristics of time and space, and are affected by topographic conditions and seasonal changes. Therefore, tourists’ viewing experience has the effect of time and space. In the layout of time and space, the viewing level of the island is richer, for example, from the far sea–offshore–coast–mountain–mountain peak in space, the tourists can experience different natural sceneries in time, and these advantages determine that the functions of island tourist destinations in sightseeing are more prominent [25]. Tourists’ perception of landscape reflects the interaction between human and landscape, and tourists’ identification and description of the perception of the natural landscape and cultural landscape become the basic basis for site selection of scenic spots and design of sightseeing routes [26]. As a special ecotourism destination [27], this study fully considered the significant influence of mountain terrain characteristics on tourists’ tour experience [28], and added accessibility factors interacting with tourists in the selection of scenic spots and route design, instead of a single terrain such as slope, aspect, and altitude. Island tourism destination planning should pay attention to the identification of marine and meteorological landscapes and the temporal and spatial combination of other landscapes, and the viewing and route location should be designed according to the characteristics of the island.

As an ecotourism destination, the island has a fragile ecological environment, which affects the sustainable development of island tourism. Some scholars have introduced landscape perception sensitivity, such as the visual perception factor, ecological perception factor, terrain factor, and social and cultural characteristics, as indicators for selecting sightseeing routes in ecotourism destinations [29]. However, in these studies, only the landscape perception of land ecotourism destinations is evaluated, and the sample size of tourism landscape data needed to calculate the landscape perception is small, which leads to the impersonal evaluation results. The advantage of this study lies in combining the ecological environment characteristics of island ecotourism destinations, collecting large samples of tourism landscape data through field investigation, providing accurate spatial positioning information and fully describing the landscape perception of island ecotourism destinations. Compared with the previous research methods of landscape perception, this paper comprehensively considers the influence of different landscape types and transportation costs on the internal accessibility of island tourism destinations, and at the same time, it uses the minimum cost distance model to make the route selection more objective and accurate. Finally, the calculation results of this paper have the characteristics of direct measurement and actual measurement verification, while other research methods mainly indirectly verify the results through the correlation with other indicators such as landscape attraction. It has been proved in the field that this method can more accurately reflect the objectivity of the actual scenic spot location and route selection.

Due to the particularity of the geographical location of the island, its ecological environment is fragile, and the rapid development of tourism will bring a series of problems to the ecological environment of the island [30,31,32]. The particularity of the island’s geographical location, the regionality of tourism resources, the fragility of the ecosystem, and other conditions restrict the sustainable development of island tourism [33]. Some scholars have evaluated the sustainable development of tourism in Nan’ao Island based on the ecological footprint. The research results show that the proportion of tourism traffic is high, which shows that traffic conditions have a great influence on the sustainable development of tourism in Nan’ao Island [32]. Lin and other scholars have also confirmed that climate temperature is also one of the important factors affecting the sustainable development of tourism [34,35]. Many scholars put forward that ecotourism is an important factor to realize the sustainable development of islands [36,37].

Based on the analysis of the special natural landscape and ecological environment of the island, this paper combines the landscape features of island tourism destinations such as landscape topography, accessibility, and environmental changes with tourists’ landscape perception, and builds an evaluation model of island spatiotemporal perception without considering tourists’ psychology and subjective experience, so as to evaluate the perception of different scenic spots, identify the optimal viewing position, extract the spatiotemporal differentiation characteristics of landscape perception, and realize the site selection and route optimization of scenic spots. In this study, the evaluation results of the optimal viewing position were verified by case study, and the evaluation results were consistent with the empirical research results. The case study preliminarily proved the feasibility of the model and calculation process. This method provides reference for landscape protection and tourism development planning in island-type ecotourism areas, and also provides new ideas and methods for the design of sightseeing routes in other island tourism development and planning projects, enriching the existing theory and method system of island tourism planning, and providing reference for the development of coastal, island, and marine tourism.

The contributions of this paper include two main points:

(1) The seasonal effect on island tourism is significant. Considering the uniqueness of island tourism development and the impact of climate conditions on tourism, this studies selects view field factors, accessibility factors, and seasonal variation factors from the perspective of time and space to analyze the spatiotemporal characteristics of a landscape, and constructs the spatiotemporal differentiation characteristics of landscape perception as the basis for site selection and route optimization of scenic viewpoints.
(2) Through the construction of a spatiotemporal perception model of the landscape, it is proposed that different observation platforms and sightseeing routes should be designed according to various types of scenic viewpoints, and tourists should be guided to carry out different types of sensory experiences on a tour. These options provide new ideas and methods for island tourism planning, especially island scenic viewpoint and sightseeing route design. This method can provide an effective reference for island tourism landscape protection, tourism development planning, and other aspects, and enrich existing island tourism planning methods.

Although the research has achieved some results, based on the spatiotemporal perception model, the perception of different scenic spots is evaluated in a more objective way, and the method of scenic spot analysis and route design for island tourism is put forward, but this method is not perfect. The method adopted in this paper is based on the only calculation of the influence of tourist terrain conditions, accessibility, and landscape changes on tourists’ landscape perception, without considering the difference in the attractiveness of landscape and ecological quality in tourists and tourists’ subjective experience. The disadvantage of this paper is that, when discussing the layout of island scenic routes, only the lowest-cost spatial analysis method is used for route planning. In future research, a particle swarm optimization algorithm or other path optimization methods can be used for in-depth analysis of route planning.

## 5. Conclusions

In this paper, Nan’ao Island is taken as an example to establish a model for the evaluation of the spatiotemporal impact factors of island landscape viewing, comprehensively applying various GIS spatial analysis methods to calculate the spatiotemporal impact factors of landscape, and preliminarily proving the feasibility of the landscape spatiotemporal perception factor model and calculation process. Based on the characteristics of landscape visual perception, a spatiotemporal perception factor model of the landscape was constructed from the perspectives of landscape topographic conditions, accessibility, and seasonal changes. According to the changing characteristics of landscape spatial location and time, the spatial accessibility factor, the best viewing time factor, and the factors influencing visual perception, such as the degree of visual field opening and the degree of visual richness, several marked landscapes were selected. A calculation function of landscape perception degree in time and space was established to quantitatively describe tourists’ perception of a landscape in different regions and periods in ecotourism areas. This can effectively identify the characteristics of spatiotemporal differentiation of landscape perception degree. Through the case study of Nan’ao Island, the best position for viewing an integrated natural landscape against the sky was identified effectively. The matching of various visual landscape types to different most scenic viewpoints was established, and the organization of island tourism routes with the most scenic viewpoints as the focus is put forward.

## Figures and Tables

**Table 1:** Spatial characteristics of scenic viewpoints in the study area.

Terrain Factors	Spatial Feature Description
Elevation	The higher the viewing position, the wider the field of view and the better the specific observation effect. The natural commanding point, namely, the top of the mountain, has the highest visibility. The omnidirectional vision and the view space are scattered.
Fluctuation	Undulation is an important factor for tourists to view the scenery, as well as a key factor for mountain building site selection and road route selection.
Slope	The larger the slope of the landscape surface relative to the viewer’s line of sight, the more likely the landscape is to be seen and noticed [19]. The scenic viewpoints should be arranged on gentle slope areas: a slope less than 15° is a candidate site for viewing.
Aspect	Sunshine duration is an important issue to consider when viewing the landscape, which is mainly related to the solar declination, latitude, slope, and aspect of the slope [20]. Generally, the shallower the slope, the longer the sunshine duration; and the steeper the slope, the shorter the sunshine duration [21]. In order to get as much sunshine as possible, the viewing position should be arranged on the south slope and southeast (southwest) slope, avoiding the north-facing back sunny slope. Taking 0° due north and 0–360° clockwise to observe the sea and land and avoid direct light, the optimal slope directions are 315–360° and 30–70° [22].

**Table 2:** Time cost values of different spatial objects.

Space Object	Road			Water Area	Landless Roadless Areas
	Provincial Road	County Road	Other Road		
Speed (km/h)	50	30	20	1	5
Time cost (min/km)	1.2	2	3	60	12

**Table 3:** Time cost values of different topographic features.

	Slope/°				Relief Degree/m			
	<5	5–15	15–25	>25	<15	15–30	30–60	>60
Speed (km/h)	5	3.3	2	1.2	5	4	3.3	2
Time cost (min/km)	12	18	30	50	12	15	18	30

**Table 4:** Weight coefficient of spatiotemporal perceptual factor model.

Target Layer	Feature Layer	Weight	Evaluation Layer	Weight
Spatiotemporal perceptual factor model	Visual landscape quality	0.527	Wide field of vision	0.324
			Visual richness	0.141
			Number of landmark landscapes	0.062
	Accessibility	0.239	Ground feature factor	0.136
			Terrain factor	0.103
	Conditions for observing the sun	0.332	Sunrise observation	0.166
			Sunset observation	0.166

**Table 5:** Types of landmark landscapes observed in sample plots.

Landscape Type	Landscape	Number of Scenic Viewpoints
Forest, farmland	Banling Orchard, Guolao Mountain Forest, Huanghua Mountain Forest Farm	1, 2, 3, 4, 5, 12, 15, 18, 19, 20, 22, 24, 28
Farmland	Banling Shanju Farm, paddy fields, terraced landscape of Yuan Mountain Village	1, 2, 3, 4, 5, 12, 15, 18, 19, 22, 24, 28
Strange and pictographic rocks	Dajianshan stone group, Dajianshan pictographic stone, Sea Stone, Jinjiaoyi pictographic stone group, Jiujian Mountain stone, Jiujian Mountain Stone Forest, Tianzi’ao stone, Fairy Stone, Eagle Stone	1, 4, 6, 9, 10, 11, 12, 16, 17, 20, 21, 22, 23, 24
Bridge	Nan’ao bridge	7,25,26
A-frame	Fengyu lighthouse, lamppost of Changshanwei Wharf	7, 8, 17, 21, 26
Pond	Guicheng Mountain pond	1,2
Cultural venues	Guicheng Square, Sailing Square	1, 2, 3, 7, 26
Small reef	Anzai Island, Beisan Island, Guan Island, Ta Island, Xiafei Island	1, 2, 4, 5, 6, 12, 13, 14, 15, 16, 18, 19, 20, 22, 24, 27, 28
Image marker	Qianjiangwan Landmark, Nan’ao Visitor Center	4, 22, 24
Tidal surge and breaking phenomenon	Tianzaiao surge	6
Recreation area	Houjiang Bay, Qianjiang Bay	4, 13, 18, 19, 22, 24, 27, 28
Recreational lakes	Huanghua Mountain Reservoir	18

**Table 6:** Timetable of sunrise and sunset positions in different seasons.

Season	Time of Sunrise	Serial Number of the Sunrise Sample Sites	Time of Sunset	Serial Number of the Sunset Sample Sites
Spring (February–April)	5:39:21–6:52:17	1, 2, 3, 4, 16, 18, 19, 28, 24, 13, 6, 8	17:58:27–18:39:31	14, 25, 20, 21, 10, 7, 26, 8, 17, 23
Summer (May–July)	5:24:47–5:41:21	18, 1, 2, 28, 19, 4, 6, 13	18:39:58–19:02:27	14, 21, 25, 7, 26, 20
Autumn (August–October)	5:42:13–6:15:22	18, 1, 2, 28, 19, 6	17:35:58–18:54:14	14, 21, 7, 26, 25
Winter (November–January)	6:17:34–6:55:42	18, 1, 2, 28, 8	17:24:55–17:55:42	25, 8, 17, 21, 7, 26

**Table 7:** Visual landscape classification of scenic viewpoints.

Types	Visual Landscape Features	Sample Number
Watch the sun and sea	View farmland + view bridge + view island + view sea, enjoy sunrise and sunset all year round, rich landscape levels, broad vision, easy to reach	26, 18, 28, 25, 7
Watch the mountains and the coast	View farmland + view forest + view island + view tidal surge, spring, summer, and autumn three seasons considerable sunrise, rich landscape level, broad vision, easy to reach	6, 5, 3, 15
Watch the mountains and the lakes	View reservoir + view forest + view stone + view island + view the sea, the year-round considerable sunrise and sunset, landscape level is general, the field of vision is wider, not easy to reach	2, 21, 19
Watch unusual or pictographic rocks and vegetation	View the strange stone + view the forest + view the island, spring and summer considerable sunset, landscape level is general, the vision is very broad, not easy to reach	20
